# Over-expression of the special AT rich sequence binding protein 1 (SATB1) promotes the progression of nasopharyngeal carcinoma: association with EBV LMP-1 expression

**DOI:** 10.1186/1479-5876-11-217

**Published:** 2013-09-18

**Authors:** Zhihua Shen, Yumei Zeng, Junli Guo, Yanxia Wu, Xiaofan Jiang, Ranran Ding, Changli Wu, Rujia Li, Botao Luo, Chao Zeng, Hanguo Jiang, Wei Jie

**Affiliations:** 1 Department of Pathology & Pathophysiology, School of Basic Medicine Science, Guangdong Medical College, Zhanjiang 524023, PR China; 2 Department of Pathology, People’s Hospital of Zhongshan City, Zhongshan 528400, PR China; 3 Hainan Provincial Key Laboratory of Carcinogenesis and Intervention, Hainan Medical College, Haikou 571199, PR China

**Keywords:** Nasopharyngeal carcinoma, Special AT rich sequence binding protein 1, Epstein Barr Virus-encoded latent membrane protein 1, Progression, Proliferation, Migration

## Abstract

**Background:**

Special AT rich sequence binding protein 1 (SATB1) plays a crucial role in the biology of various types of human cancer. However, the role of SATB1 in human nasopharyngeal carcinoma (NPC) remains unknown. In the present study, we sought to investigate the contribution of aberrant SATB1 expression in the progression of NPC and its association with the Epstein Barr virus (EBV)-encoded latent membrane protein 1 (LMP-1).

**Methods:**

Immunohistochemical analysis was performed to detect SATB1 and LMP-1 protein in clinical samples, and the association of SATB1 protein expression with patient clinicopathological characteristics and LMP-1 expression were analyzed. SATB1 expression profiles were evaluated in well-differentiated NPC cell line CNE1, poorly-differentiated CNE2Z, undifferentiated C666-1 and immortalized nasopharyngeal epithelia NP-69 cells using quantitative RT-PCR, western blotting and fluorescent staining. After inhibition the SATB1 expression by using SATB1 specific small interfering RNA in these cell lines, the change of cell proliferation was investigated by western blotting analysis of PCNA (proliferating cell nuclear antigen) expression and CCK-8 assay, and the cell migration was assessed by Transwell migration assay. Finally, the expressions of SATB1 and PCNA were examined in CNE1 cells that forced LMP-1 expression by fluorescent staining and RT-PCR.

**Results:**

Immunohistochemical analysis revealed that SATB1 protein expression was elevated in NPC tissues compared to benign nasopharyngeal tissues (*P* = 0.005). Moreover, high levels of SATB1 protein expression were positively correlated with clinical stage (*P* = 0.025), the status of lymph node metastasis (N classification) (*P* = 0.018), distant metastasis (M classification) (*P* = 0.041) and LMP-1 expression status (*r* = 2.35, *P* < 0.01) in NPC patients. *In vitro* experiments demonstrated that an inverse relationship between SATB1 expression and NPC differentiation status, with SATB1 weakly expressed in NP-69 cells and CNE1 cells, and significant increasingly expressed in CNE-2Z and C666-1 cells. Targeted knockdown of SATB1 expression obviously attenuated the proliferation and migration of highly SATB1-expressing CNE2Z and C666-1 cells, but not NP-69 and CNE1 cells. Interestingly, forced LMP-1 expression in CNE1 cells led to a surprisingly increasing SATB1 expression and nuclear location, companying with an up-regulated PCNA expression.

**Conclusions:**

Our results reveal that EBV LMP-1-mediated over-expression of SATB1 is associated with NPC progression, suggesting SATB1 may represent a promising biomarker and therapeutic target for NPC.

## Background

The nuclear factor special AT-rich sequence binding protein 1 (SATB1), which was cloned in 1992, is considered to be a global chromatin organizer [1]. By providing a nuclear architectural platform, SATB1 regulates gene expression by promoting chromatin remodeling and regulating the levels of histone acetylation and methylation [2,3]. Under normal physiological conditions, SATB1 is highly expressed in the thymus, with low levels also present in the testes, fetal brain and osteoblasts; however, expression is virtually undetectable in other tissues [4,5]. SATB1-null mice display abnormal expression of numerous genes involved in T-cell development [5], implying a critical role for SATB1 in the maintenance of the immune system.

Recently, accumulating data suggests that over-expression of SATB1 contributes to tumor progression [6]. Studies by Han *et al.* revealed that SATB1 is over-expressed in aggressive breast cancer cells and the level of SATB1 expression had high prognostic significance. In this study, targeted silencing of SATB1 led to altered expression of >1000 genes, predominantly controlling cell adhesion, signaling, extracellular matrix formation and cell cycle [7]. This report opened a new window regarding the role of SATB1 in tumor biology, despite controversial studies [8,9]. To date, high expression of SATB1 has been linked to the progression of human gastric cancer [10-12], ovarian carcinoma [13], liver cancer [14-16], rectal cancer [17,18], laryngeal squamous cell carcinoma [19], cutaneous malignant melanoma [20], and prostate cancer [21,22]. In contrast to these reports, however, Selinger *et al.* reported that loss of SATB1 is associated with poor prognosis in lung squamous cell carcinoma [23]. Thus, the role of SATB1 in tumorigenesis remains unclear and requires further investigation.

Nasopharyngeal carcinoma (NPC) is a malignant cancer derived from the nasopharyngeal epithelium. Over the past decades, epidemiological studies have shown that NPC appeared the unique prevalence features, including regional, racial familial aggregation and Epstein-Barr virus (EBV) infection status. NPC is one of the most common malignant diseases in Chinese and persons of south-east Asian descent, and the provinces of Guangdong, Guangxi, Hainan, Hunan and Fujian are the five most endemic areas in China [24]. Histologically, non-keratinizing carcinoma accounts for >97% of NPC cases in endemic areas [25]. Clinically, most NPC patients present with cervical lymphadenopathy at primary diagnosis [26], which represents a more advanced stage. Therefore, current approaches aimed at improving the rate of early detection, diagnosis and treatment in NPC patients, deserves significant attention.

NPC is an EBV associated carcinoma, and EBV-encoded oncogene latent membrane protein 1 (LMP-1) contributes to the carcinogenesis of NPC. Previous studies in our department focused on the role of the LMP-1 in the pathogenesis of NPC [27-31]. In the present study, we aimed to clarify the role of aberrant SATB1 expression in NPC progression. We investigated the correlation between SATB1 protein expressions with NPC patient clinicopathologic parameters. Furthermore, we analyzed the expression of SATB1 in a panel of NPC cell lines characterized by varied differentiation and LMP-1 expression status. Using RNA interference (RNAi), we investigated the effect of targeted knockdown of SATB1 on NPC cell proliferation and migration. Finally, the expression of LMP-1 and its association with SATB1 expression in NPC were investigated.

## Methods

### Patients and specimens

Clinical, paraffin-embedded samples were obtained from 119 patients from the Affiliated Hospital of the Guangdong Medical College (Zhanjiang, Guangdong, China) and the People’s Hospital of Zhongshan City (Zhongshan, Guangdong, China). All patients did not receive preoperative radiotherapy or chemotherapy. The use of the human tissue in this study was approved by the Ethics Council of the Affiliated Hospital of the Guangdong Medical College and the People’s Hospital of Zhongshan City for Approval of Research Involving Human Subjects. Subjects comprised NPCs (*n* = 95; 71 men, 24 women) and nasopharyngeal epithelial hyperplasia (NEH, *n* = 24; 18 men, 6 women). Clinical data of patients was reviewed based on the pathology tumor–node–metastasis (pTNM) system (AJCC⁄UICC 2002). Among 119 cases, the clinicopathologic variables of 115 patients were shown as described previously [27], and the remained 4 cases were newly involved. NPC patients were diagnosed for the first time at an average age of 42.9 years (range, 23–72 years). There were 5 samples in stage I, 10 samples in stage II, 36 samples in stage III, and 44 samples in stage IV. All NPC patients were diagnosed with non-keratinizing carcinoma following histological examination.

### Immunohistochemistry (IHC)

IHC was performed using monoclonal antibodies against SATB1 (Cat. #2938-1, 1/100; Epitomics, Burlingame, CA, USA) and LMP-1 (CS1-4, 1:50; DAKO Glostrup, Denmark). The process of IHC was performed as stranded protocols. Briefly, paraffin sections (5 μm) were prepared for immunohistochemical staining by heating sections in sodium citrate buffer (pH 6.0, 10 mM), and endogenous peroxidases were blocked by incubation in 0.3% H_2_O_2_. Sections then were incubated with primary antibodies at 4°C overnight and non-immune IgG was used as negative control. Antigenic sites were localized using a SP9000 kit and 3,3′-diaminobenzidine (DAB) kit (ZSGB-BIO, Beijing, China). The immunoreactive score (IRS) of SATB1 was calculated as previously described [17,28]. In brief, the staining intensity was classed as 0, negative; 1, weak; 2, moderate and 3, strong. The percentage of SATB1-positive cells was scored as 1, 0 – 9% positive cells; 2, 10 – 50% positive cells and 3, >50% positive cells. Samples with a sum IRS <6 were considered to display low expression of SATB1, and samples with a sum IRS ≥6 were considered to display high expression of SATB1. Scoring system for LMP-1 was done according to Khabir *et al*. [29]. Briefly, immunostaining intensity was rated as follows: 0, none; 1, weak; 2, moderate; and 3, intense. The percentage of LMP-1-positive cells was score as 0, none seen in the section; 1, 0 – 25% positive cells; 2, 26 – 50% positive cells; 3, 51 – 75%; and 4, 76 – 100%. In the present study, sections with a sum IRS ≥1 were considered to be LMP-1 positive.

### Cell culture

Well-differentiated NPC cell line CNE1 (LMP-1-), CNE1-GL (ectopic expression of EBV-LMP-1 in CNE1 cells, LMP-1+), poorly-differentiated CNE2Z (LMP-1-) and undifferentiated C666-1(LMP-1+) and the immortalized nasopharyngeal epithelial cell line NP-69 (LMP-1-) were maintained as previously described [27,30]. Briefly, CNE1, CNE2Z and C666-1 cell lines were cultured in Dulbecco’s modified Eagle’s medium (DMEM, Thermo Scientific, Beijing, China) supplemented with 10% heat-inactivated fetal bovine serum (FBS) (Thermo Scientific), 100 U/ml penicillin and 100 μg/ml streptomycin (Solarbio Science & Technology Co., Ltd, Beijing, China). CNE1-GL cell line was maintained in DMEM containing 10% FBS, 100 U/ml penicillin, 100 μg/ml streptomycin and 1 μg/ml puromycin. The immortalized NP-69 was cultured in defined keratinocyte serum-free medium (cat. #10744-019, Invitrogen, Life Technologies, Guangzhou, China) supplemented with 5% heat-inactivated FBS, 100 U/ml penicillin, 100 μg/ml streptomycin, and 0.2 ng/ml recombinant epidermal growth factor. All cell lines were cultured at 37°C in a humidified atmosphere with 5% CO_2_.

### RNA interference (RNAi)

Small interfering RNAs (siRNAs) were purchased from RiboBio Co., Ltd. (Guangzhou, China). For RNAi experiments, a cocktail of two pairs of independent siRNAs targeting the *SATB1*(Accession: NM_002971.4) coding region were used; si-SATB1-1 forward, 5′-GGAUAGUCUUUCUGAGCUAdTdT-3′, si-SATB1-1 reverse, 5′-UAGCUCAGAAAGACUAUCCdTdT-3′; si-SATB1-2 forward, 5′-GCU GAAAGAGACCGAAUAUdTdT-3′, si-SATB1-2 reverse, 5′-AUAUUCGGUCUC UUUCAGCdTdT-3′. A corresponding scrambled sequence (si-Control, Cat. #siB05815) was used as a negative control. One day prior to transfection, CNE1, CNE2Z, C666-1 and NP-69 cells were seeded in 6-, 24- or 96-well plates supplemented with complete medium without antibiotics. Sub-confluent (60-70%) cells were transfected with siRNAs using Lipofectamine™ 2000 (Invitrogen) in Opti-MEM medium (Invitrogen). Following incubation of cells at 37°C in a humidified atmosphere of 5% CO_2_ for 6 hours, media were replaced with complete cell culture media. Transfection efficiency was confirmed by fluorescence microscopy of cells transfected with Cy3-conjugated control oligos. The cells were harvested at different time points after transfection for the following experiments.

### RNA extraction and reverse transcriptase PCR (RT-PCR)

Total RNA was extracted with Trizol reagent (Invitrogen, Carlsbad, CA, USA). cDNA was synthesized using 1 μg of total RNA and the to generate the first strand cDNA by oligo(dT) 18 using the Fermentas RT System (Cat. #K1622, Thermo Scientific). The levels of LMP-1 and SATB1 transcrtipts in various NPC cell lines were assessed by semiquantitative PCR or quantitative PCR, respectively. The primer pairs used for PCR were included in the Additional file 1. Semiquantitative PCR was performed with Mastercycler® Gradient Thermal cycler (Eppendorf, Germany) at the annealing tempreture of 55°C with total 32 cycles. Products of PCR were separated by 1.5% agarose gel electrophoresis and visualized under UV using InGenius LHR gel Documentation and analysis system (InGene, Fredrick, MO, USA). Quantitative PCR was conducted using the LightCycler480 instrument (Roche (China) Ltd., Shanghai, China) in a final volume of 20 μl, including 10 μl SYBR Green I PCR Master Mix (TOYOBO, OSAKA, Japan), 0.4 μl forward primer (10 μM), 0.4 μl reverse primer (10 μM), 2 μl cDNA and 7.2 μl dH_2_O. PCR amplification was performed as follows; 95°C for 1 minute, then 40 cycles of 95°C for 15 seconds, 60°C for 1 minute. The relative abundance of *SATB1* mRNAs were determined from the C_T_ values and plotted as the fold change compared with the control groups. For all PCR analysis, the transcription levels of β-actin were served as a loading controls.

### Western blotting

Cells were collected and lysed with RIPA lyses buffer (Cat. # P0013C, Beyotime Institute of Biotechnology, Jiangsu, China). 30–40 μg total proteins were subjected to sodium dodecyl sulfate polyacrylamide gel electrophoresis (SDS-PAGE), and then proteins were transferred to the polyvinylidene difluoride (PVDF) membranes (0.22 μM pore size). After twice washed with TBST, the membranes were incubated with 5% skimmed milk in TBST at 37°C for 1 hour, then the membrane were incubated with the primary antibodies (SATB1, 1/500, Epitomics; Proliferating cell nuclear antigen (PCNA), 1/400, Santa Cruz, CA, USA; LMP-1, 1/400, DAKO Glostrup, Denmark; β-actin, 1/1000, Santa Cruz) at 4°C overnight, After twice washed by TBST, the membranes were incubated with horseradish peroxidase (HRP)-conjugated secondary antibodies for 1 hour at 37°C. Bands were visualized using enhanced chemiluminescence (ECL) reagents (Thermo Fisher, Rockford, IL, USA) and analyzed with gel analysis system (BIO-RAD VersDoc TM5000MP System, Guangzhou, China). The expressions of β-actin were used as loading control.

### Immunofluorescence staining

Indirect immunofluorescence was performed on cells grown on glass coverslips for 48 hours. Cells were incubated overnight with primary SATB1 (rabbit-anti, 1/100; Epitomics), PCNA (mouse-anti, 1/100; Cell Signaling Technology, Inc., Danvers, MA, USA) and LMP-1 (mouse-anti, 1/50; DAKO Glostrup, Denmark) antibodies at 4°C. After twice washed by 1 × PBS, antigenic sites were subsequently localized using FITC- or TRITC-conjugated goat anti-rabbit or -mouse IgG (1/100, Protein Tech Group, Inc., Chicago, IL, USA). Diamidino-2- phenylindole (DAPI) was used to stain the nuclei. Images were captured using a laser scanning confocal microscope (TCS SP5, Leica, Germany).

### In vitro cell proliferation assay

Cell proliferation was assessed 24 hours and 48 hours post-transfection with siRNAs using the Cell Counting Kit-8 (CCK-8) assay, in accordance with the manufacturer’s instructions (Biyuntian, Jiangsu, China). Briefly, transfected cells (4 × 10^3^ per well) were seeded in 96-well plates and cultured for either 24 hours or 48 hours, after washed with 1 × PBS, 10 μl CCK-8 reagent plus 100 μl basal DMEM medium was added per well and incubated at 37°C for 2 hours. The optical density (OD) was subsequently measured using a microplate reader (Multiskan MKS, Thermo Scientific, Waltham, MA, USA) by dual wavelength mode (450/630 nm). Data represent the mean ± SD of three independent experiments.

### Transwell migration assay

In vitro cell migration assays were performed as previously described [30] using Transwell chambers (8-μm polycarbonate membranes, BD Biosciences). In brief, 48 hours following transfection with si-SATB1, si-Control or no transfection, cells were harvested in DMEM supplemented with 0.5% FBS. 2 × 10^4^ cells were seeded in the upper chamber which was placed over the lower chamber containing DMEM medium supplemented with 10% FBS. Migration was allowed to proceed for 12 hours at 37°C, and insert membranes were fixed for 20 minutes with 70% ethanol, and stained with 0.5% Eosin. Membranes were washed with PBS and cut from the inserts. Cells on the upper surface of the membrane were removed with a cotton swab and membranes were mounted with glycerol. The number of migrated cells on the lower surfaces of the membranes was determined by counting fifteen representative fields from triplicate inserts.

### Statistical analyses

Statistical analyses were performed using PRISM Software (GraphPad Software, CA, USA). χ ^2^ and continuity corrected χ ^2^ test (when cells have expected count less than 5) were used to analyze the comparisons of SATB1 and LMP-1 expression in clinical samples; Data of in vitro experiments were expressed as the mean ± SD. For analysis of differences between two groups, Student’s *t*-test was performed. For multiple groups, ANOVA was performed followed by the Student–Newman–Keuls test. *P* values <0.05 were considered statistically significant.

## Results

### Over-expression of SATB1 protein is correlated with clinicopathological features of human NPC

**Figure 1:**
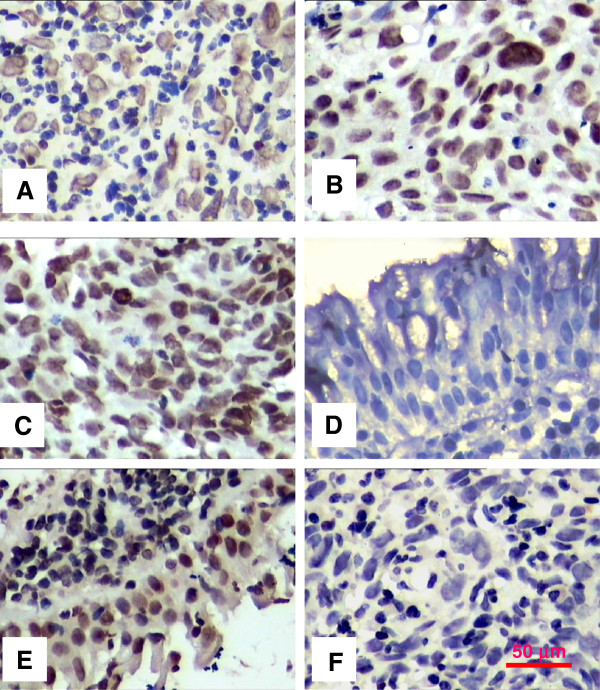
**Representative photographs of SATB1 expression in samples of nasopharyngeal carcinoma (NPC) and non-cancerous nasopharyngeal epithelial hyperplasia (NEH) were detected by immunohistochemistry.** The immunohistochemical PV9000 method was used to detect SATB1 protein expression in clinical samples. Non-immune IgG was used as a negative control. The expression and location of SATB1 in cells was colorized with DAB and counterstained with hematoxylin. **(A)**: IRS <6.0 in NPC. **(B)** &**(C)**: IRS >6.0 in NPC. **(D)**: IRS <6.0 in NEH. **(E)**: IRS >6.0 in NEH. **(F)**: IgG control. Original magnification, 400×. Scale bars = 50 μM.

SATB1 protein expression was analyzed by immunohistochemistry in tissues from 24 patients with NEH and 95 patients with NPC. High expression of SATB1 protein (IRS ≥6) was observed in the nucleus of 65.3% of NPCs (62/95), compared to 33.3% (8/24) of patients with NEH (*P* = 0.005) (Table 1, Figure 1), suggesting a crucial role for SATB1 over-expression in NPC pathogenesis. We next analyzed the association of SATB1 expression with the clinical parameters of NPC patients. While no correlation was observed between SATB1 high expression and patient gender, age or smoking condition, a positive correlation with clinical classification (I-II *vs* III-IV**,***P* = 0.025), N classification (N0 *vs* N1-N3, *P* = 0.018) and M classification (M0 *vs* M1, *P* = 0.041) was observed (Table 1). We also observed an association between SATB1 expression and T classification (T1-T2 *vs* T3-T4, *P* = 0.077), although this was not statistically significant. Together these results strongly indicate that high expression of SATB1 protein contributes to the clinical progression of human NPC.

**Table 1:** Correlation between the clinicopathologic characteristics and expression of SATB1 protein in NPC and NEH

**Clinical parameters**	***n***	**SATB1 expression**		**χ **^**2**^	***P *value**
		**high**	**low**		
**Histological types**					
NPC	95	62	33	8.065	0.005*
NEH	24	8	16		
**Smoking**					
Yes	45	30	15	0.743	0.785
No	50	32	18		
**Gender**					
Male	71	48	23	0.680	0.410
Female	24	14	10		
**Age**					
≧50	46	27	19	1.697	0.193
< 50	49	35	14		
**Clinical classification**					
I-II	15	6	9	5.015	0.025*
III- IV	80	56	24		
**T classification**					
T1 - T2	32	17	15	3.136	0.077
T3 - T4	63	45	18		
**N classification**					
N0	19	8	11	5.618	0.018*
N1-N3	76	54	22		
**M classification**					
M0	81	49	32	4.180^Δ^	0.041*
M1	14	13	1		

NEH: nasopharyngeal epithelial hyperplasia, ^Δ^ Continuity corrected *χ*^2^ test.

* Significance as indicated.

### Expression features of SATB1 in NPC and NP-69 cell lines

**Figure 2:**
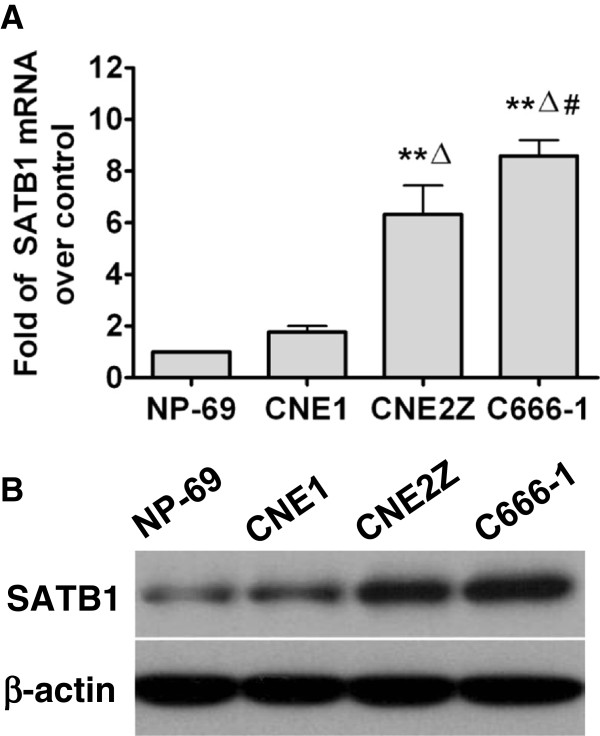
**Expression of SATB1 mRNA and protein in various types of NPC and immortalized NP-69 cell lines. (A)**: Well-differentiated CNE1 cells, poorly- differentiated CNE2Z cells, undifferentiated C666-1 cells and the immortalized non-cancerous NP-69 cells were seeded in 24-well plates (*n* = 3) and harvested for analysis of *SATB1* expression by quantitative reverse transcription polymerase chain reaction (qRT-PCR). *β-actin* was used as a loading control. ** *P* <0.01 vs NP-69, △*P *<0.05 vs CNE1, # *P* <0.05 vs CNE2Z. **(B)**: Western blotting was used to detected SATB1 protein expression in CNE1, CNE2Z, C666-1 and NP-69 cell lines. β-actin was used as a loading control.

**Figure 3:**
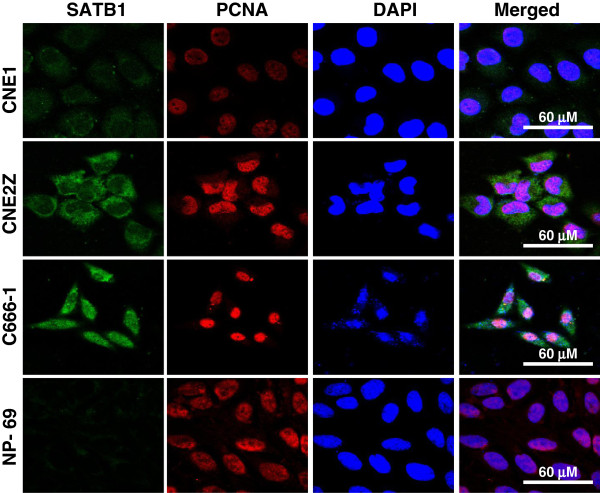
**Representative fluorescence photographs of SATB1 and PCNA protein expression in various NPC cell lines and NP-69 cells.** Cells were grown on glass coverslips for 48 hours and incubated with primary rabbit-anti human SATB1 and mouse-anti human PCNA antibodies overnight. The antigenic sites of SATB1 and PCNA were detected using FITC-, TRITC-conjugated goat anti-rabbit and anti-mouse IgG and analyzed by confocal microscopy. And cells were countstained with DAPI. Scale bars = 60 μM.

To further examine the relationship between SATB1 expression and NPC pathogenesis, we analyzed the expression of SATB1 in three cell lines representing various differentiation stages of NPC. As shown in Figures 2 and 3, the expression of SATB1 mRNA and protein varied between the different NPC cell lines. In general, we observed a very low level of SATB1 expression in immortalized NP-69 cells, which was predominantly localized in the cytoplasm. In contrast, SATB1 expression was increasingly observed in well-differentiated CNE1 cells, poorly-differentiated CNE2Z cells and undifferentiated C666-1 cells. SATB1 mainly located in cytoplasm in CNE1 and CNE2Z cells, but increasing nucleus location in C666-1 cells was observed (Figure 3). These results suggest that SATB1 expression is inversely associated with the cellular differentiation status of NPC.

### Inhibition of SATB1 attenuates NPC cell proliferation

**Figure 4:**
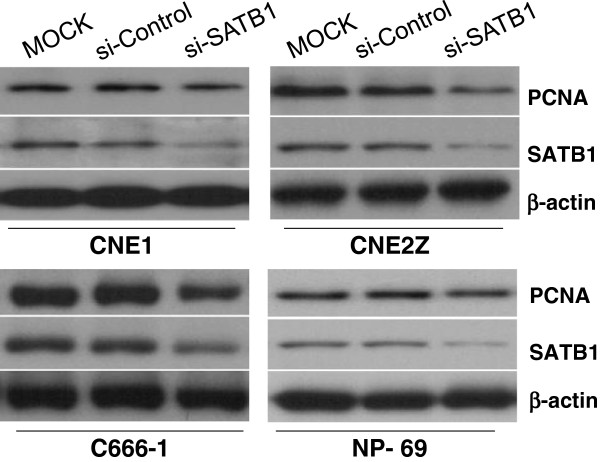
**SATB1 specific siRNA cocktails inhibit SATB1 and PCNA proteins expression.** After transfected CNE1, CNE2Z, C666-1 and NP-69 cells with a cocktails of siRNA (si-SATB1), control siRNA (si-Control) or untransfected (MOCK) for 48 hours, 40 μg total proteins were used to examine SATB1 and PCNA proteins expression in these cells by western blotting analysis. β-actin was used as a loading control.

**Figure 5:**
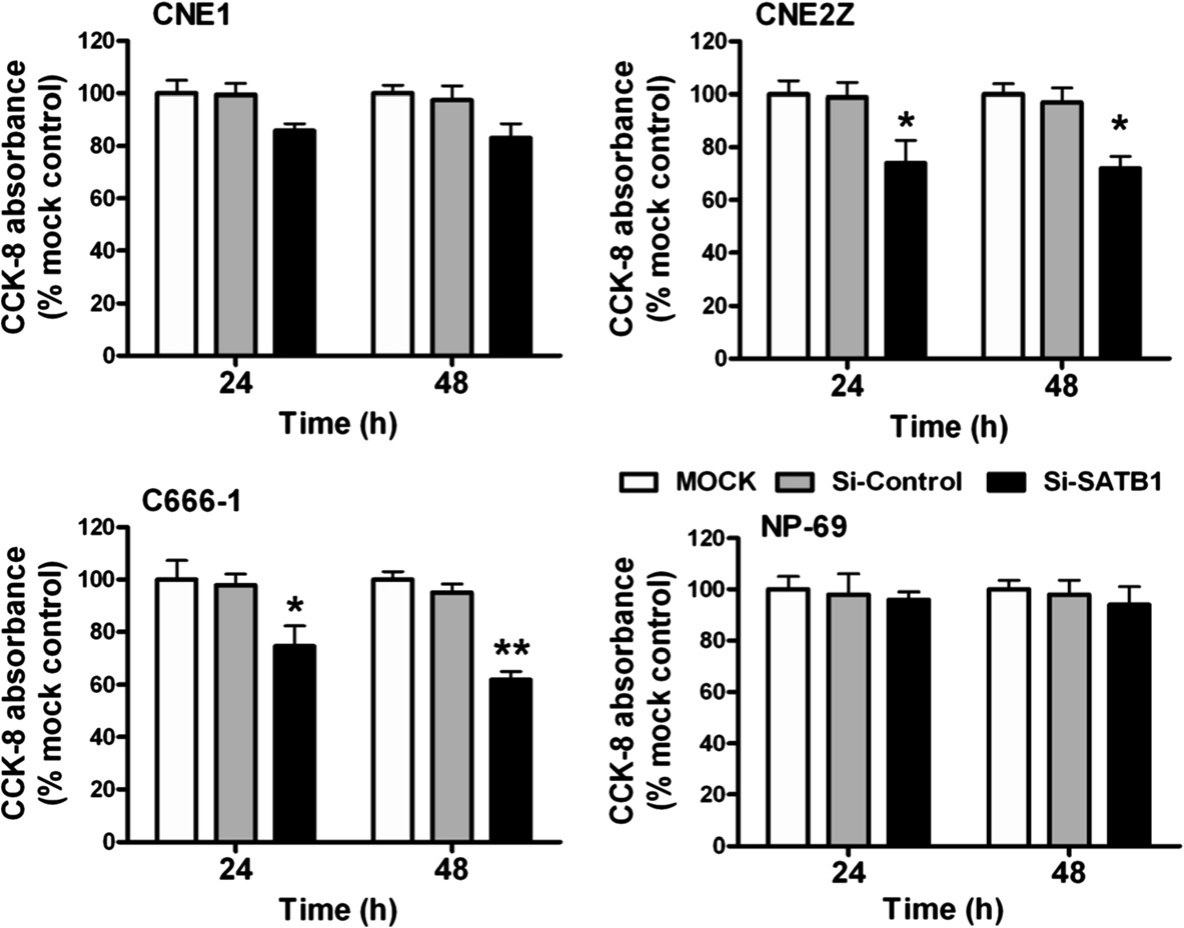
**Inhibition of SATB1 expression attenuates NPC cells proliferation.** CCK-8 Assay was used to assess cellular viability of CNE1, CNE2Z, C666-1 and NP-69 cells after 24 hours and 48 hours following transfection with cocktails of SATB1 siRNA (si-SATB1), control siRNA (si-Control) or untransfected (MOCK). Values of optical density (OD) were obtained by the absorbance at the dual wavelengths 450/630 nM, and the results indicating the cell viability were plotted as the percentage over controls (MOCK cells). * *P* <0.05, ** *P* <0.01 *vs* Mock or si-Control–treated groups.

To investigate the effect of SATB1 on NPC cell proliferation, we performed knockdown of SATB1 in C666-1 cells using three independent siRNAs targeting the SATB1 coding region. Initial testing of these siRNAs revealed that the cocktail of si-SATB1-1 and si-SATB1-2 displayed the significant degree of SATB1 knockdown in C666-1 cells (inhibitory rate >85%, data not shown), and this cocktail of two pair of siRNA oligos was therefore utilized in subsequent experiments. And the results of western blotting confirmed that transfection this siRNA cocktails to NPC cells led to a significant decrease of SATB1 protein expression, besides, attenuated PCNA protein expressions were also observed (Figure 4). Then the cell proliferation was examined by CCK-8 assays post transfection these 4 cell line with SATB1 specific siRNA cocktails. As shown in Figure 5, transfection of the NPC cell lines with this siRNA cocktails led to significantly damaged cell viability in CNE2Z and C666-1 cells, but not the CNE1 and NP-69 cells. Taken together, these results demonstrate that over-expression of SATB1 plays a crucial role in NPC cell proliferation.

### Inhibition of SATB1 represses NPC cell migration

**Figure 6:**
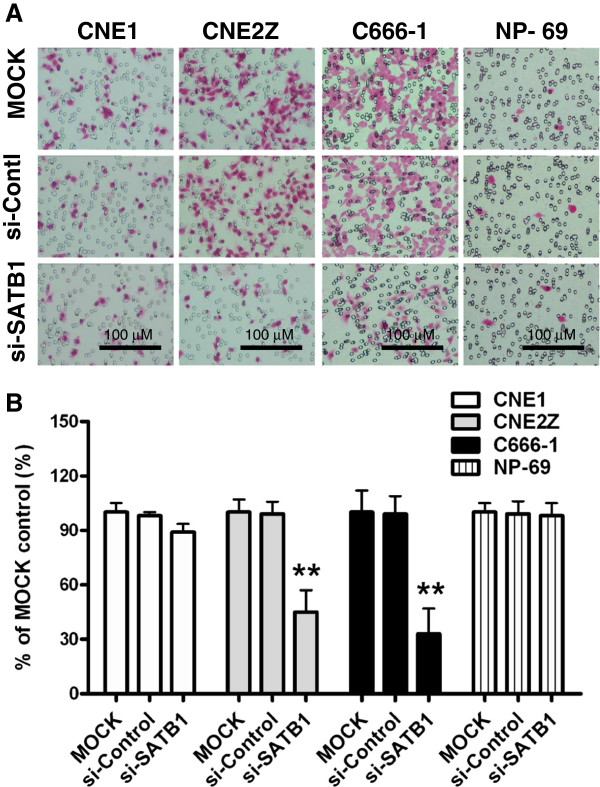
**Knock-down of SATB1 suppresses NPC cell migration.** Cell migration was assessed 48 hours following transfection of CNE1, CNE2Z, C666-1 cells and NP-69 with cocktails of SATB1 siRNA (si-SATB1), control siRNA (si-Control) or untransfected (MOCK) by Transwell migration assay. Migrated cells present on the lower surfaces of the membranes were counted within fifteen representative fields in triplicate inserts. **(A)**: Representative photographs of cell migration. **(B)**: Cell migration and percentages. the results indicating the percentage of cell migration were plotted as the percentage over controls (MOCK). **P <0.01 *vs* Mock or si-Control-treated groups. Scale bars = 100 μM.

Cervical lymph node metastasis is one of the most common clinical symptoms of NPC patients at primary diagnosis [26], therefore blocking the migration of NPC cells may represent a promising treatment strategy. Indeed, treatment of highly SATB1-expressing CNE2Z and C666-1 cells with SATB1-siRNAs led to a significant decrease in cell migration, while migration was unaffected in CNE1 cells and immortalized NP-69 cells (Figure 6). These results suggest that SATB1 over- expression in NPC cells promotes migration.

### The association between LMP -1 and SATB1 expression in NPC

**Figure 7:**
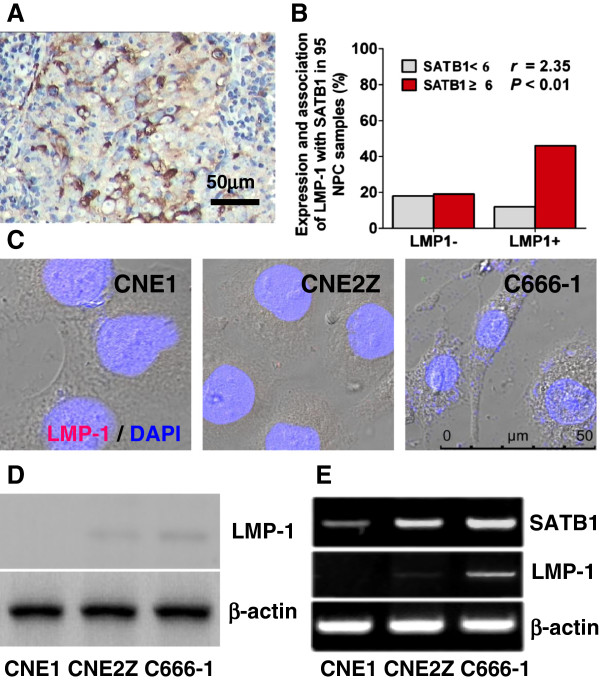
**LMP-1 expression in clinical NPC samples and NPC cell lines and its correlation to SATB1. (A)** Representative photograph of LMP-1 expression in NPC samples examined by immunohistochemistry (IRS >6.0). **(B)** Association between LMP-1 and SATB1 expression in clinical NPC samples. **(C)** Immunofluorescence staining, **(D)** Western blotting and **(E)** RT-PCR were used to detect the LMP-1 expression in well-differentiated CNE1, poorly-differentiated CNE2Z and un- differentiated C666-1 cell lines. β-actin was used as a loading control.

**Figure 8:**
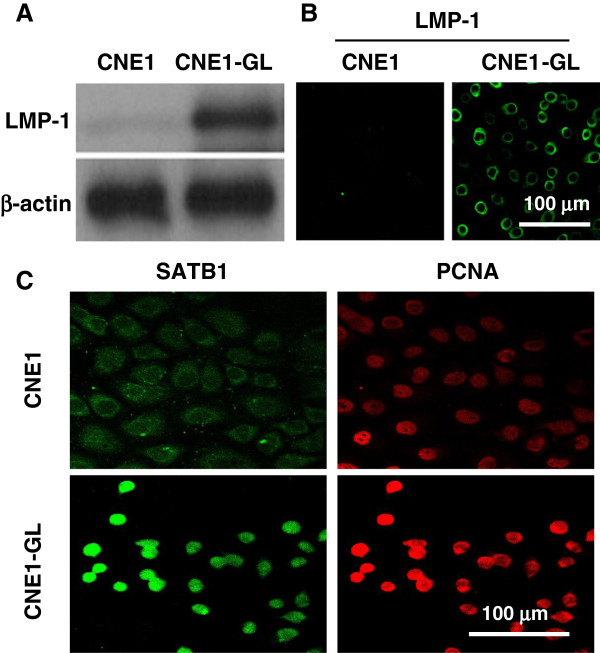
**LMP-1 induces SATB1 expression and nuclear location in NPC cells.** CNE1 (LMP1-) and CNE1-GL (LMP1+, ectopic expression of EBV LMP1 in CNE1 cells) cells were harvested and then **(A)** western blotting and **(B)** Immunofluorescence staining were used to detect LMP1 expression. **(C)** Immunofluorescence staining was used to detect SATB1 and PCNA proteins expression and its sub-cellular location. Antigens were localized by FITC- or TRITC-conjugated IgGs. Scale bars = 100 μM.

Immunohistochemistry was used to assess LMP-1 expression in 95 clinical NPC samples. In the present investigation, we just divided NPC into LMP-1 positive (LMP-1+, IRS ≥1) and LMP-1 negative (LMP-1-, IRS < 1) groups. By this ways, there were 61.1% (58/95) NPC samples showed LMP-1+; Among 58 LMP-1+ cases, 46 samples were the same SATB1 high expression (IRS ≥6). A positive correlation of LMP-1 to SATB1 expression in clinical NPC samples was appeared (*r* = 2.35, *P* < 0.01, Figure 7). Further, we performed immunflurescent staining, western blotting and RT-PCR analysis to assess LMP-1 expression in CNE1, CNE2Z and C666-1 cells lines. As results, there were no obviously LMP-1 protein expressions in three NPC cell lines, which were verified by results of immunflurescent staining and western blotting, however, the transcripts of LMP-1 could be notably detected in C666-1 cells; interestingly, LMP-1 transcripts expression levels in NPC cell lines were positively associated with SATB1 mRNA expression (Figure 7). To further investigate the relationship between LMP-1 and SATB1 in NPC cells, a LMP-1 over-expression plasmid (PAT-LMP-1-GFP) was transfected into CEN1 cells to generate LMP-1 stably expressed NPC cell line CNE1-GL [31], then we found that forced LMP-1 expression in CNE1 cells led to a surprisingly increased SATB1 expression and nuclear location, companying with the up-regulation of PCNA (Figure 8). Together these results referred that LMP-1 may mediate SATB1 expression in NPC cells.

## Discussion

SATB1 promotes tumor metastasis by providing a nuclear architectural platform regulating the expression of >1000 genes, many of which are related to cell growth and translocation [7]. In the present study, we detected over-expression of SATB1 protein (IRS ≥6) in 65.3% of NPCs (62/95), which was significantly higher than that observed in benign NEH (8/24), suggesting that SATB1 may play a role in NPC pathogenesis. We then further assessed the correlation between SATB1 protein over-expression and NPC patient clinical parameters. We observed that SATB1 protein over-expression was positively correlated with clinical stage (*P* = 0.025), the status of lymph node metastasis (N classification) (*P* = 0.018) and distant metastasis (M classification) (*P* = 0.041). These data suggest that high expression of SATB1 contributes to the clinical progression of NPC. Our results are consistent with other reports showing that high expression of SATB1 promotes cancer progression in multiple human carcinomas [7,11-14,17,19,32].

We next examined SATB1 expression in NPC cell lines at various stages of differentiation. We found that when compare with a basal levels of SATB1 expression in the immortalized NP-69 cells, the well-differentiated CNE1 cells displayed a low levels of SATB1 expression, while the highly proliferative and poorly-differentiated CEN2Z cells, and undifferentiated C666-1 cells exhibited increasing SATB1 expression and a increase of nuclear localization (Figure 2 and 3). These results indicate that the expression of SATB1 is inversely correlated to NPC cell differentiation. Our findings support the very recent report of Barboro *et al.,* demonstrating that SATB1, a DNA matrix attachment regions (MARs) binding protein, is involved in the differentiation of prostate cancer cells [33]. In the present study, SATB1 expression co-localized with PCNA, a marker of cell proliferation, in NPC cells (Figure 3). This is in accordance with a study by Chu *et al.*[34], which showed that SATB1 expression was positively correlated with Ki67 expression in gliomas.

In human epithelium-derived carcinoma cells such as breast cancer cells, inhibition of SATB1 expression by siRNA or Decoy-DNA led to reversal of cell malignancy [7,32]. In addition, knockdown of SATB1 in human non-epithelium- derived cancers such as U251 glioma cells also led to inhibition of cell growth, invasion, metastasis and angiogenesis [34]. These results highlight the possibility of molecularly targeting SATB1 in cancer treatment. In this study, we found that treatment of SATB1-expressing NPC cells with SATB1-specific siRNAs, attenuated cell proliferation and motility which verified by the changes of expression of PCNA (Figure 4), cell viability (Figure 5) and cell migration (Figure 6). Our results support the rationale that targeting SATB1 may represent an alternative therapeutic strategy in the treatment of NPC.

NPC is an EBV associated malignant carcinoma, especially in endemic area such as Southern China. Previous studies in our department focused on the role of the EBV-encoded LMP-1 in the pathogenesis of NPC [31,35-38], and the results of our lab and others have previously indicated that the LMP-1 could promote NPC progression. In the present study, our preliminary data revealed that LMP1 expression was positively associated with SATB1 expression in clinical NPC specimens (*r* = 2.35, *P* < 0.01); in addition, the expression level of SATB1 was correlation with LMP-1 expression status in NPC cell lines (Figure 7); much interestingly, ectopic expression of LMP-1 strongly promoted SATB1 expression and its nuclear location, as well as an up-regulation of PCNA, in well-differentiated CNE1 cells (Figure 8). Combining results about the SATB1 expression styles in CNE1, CNE2Z, C666-1 and CNE-GL cells and its correlation to LMP-1 expression in clinical NPC samples, it seemed that LMP-1 stimulates cell proliferation partly via up-regulating of SATB1 expression in NPC cells.

The process of epithelial-to-mesnchymal transition (EMT) is one of several key factors involved in tumor metastasis. Previous reports have shown that NPC cells have undergone the process of EMT [39-41], in particular the neoplastic spindle cells in NPC [40]. Our present results showed that knockdown of STAB1 expression by SATB1 specific siRNA led to an obvious up-regulation of E-cadherin and a down-regulation of Vinmentin, the marker for EMT (Additional file 2: Figure S1). These results therefore support previous reports regarding the role of EBV infection in EMT in NPC [41-43]. Very recently, a report of Endo K and colleagues had revealed that EBV LMP1 could induce SATB1 expression in human NPC [44]. Thus, we hypothesized that EBV may promote NPC progression partly by modulation the process of EMT via LMP1-mediated SATB1 expression.

## Conclusions

Taken together, our study provides the evidence that EBV LMP-1 expression may associate with SATB1 expression in NPC; over-expression of SATB1 is closely related to the clinical progression of NPC, SATB1 was universally expressed in all NPC cell lines examined, and its expression levels were inversely related with cell differentiation; knockdown of SATB1 by specific siRNA led to attenuated cell proliferation and migration. Our results highlight the possibility of SATB1 as a promising biomarker and therapeutic target for NPC. Future studies will focus on i) factors such as LMP-1 controlling SATB1 expression in the NPC cells, ii) the combination of factors working in concert with SATB1 in NPC cells, and iii) investigating how SATB1 mediates the process of EMT in NPC cells.

## Abbreviations

CCK-8: Cell Counting Kit-8; DAB: 3,3'-diaminobenzidine; DMEM: Dulbecco’s modified Eagle’s medium; EBV: Epstein-Barr virus; ECL: Enhanced chemiluminescence; EMT: Epithelial-to-mesnchymal transition; FBS: Fetal bovine serum; HRP: Horseradish peroxidase; IHC: Immunohistochemistry; IRS: Immunoreactive score; LMP1: Latent membrane protein 1; MARs: Matrix attachment regions; NEH: Nasopharyngeal epithelial hyperplasia; NPC: Nasopharyngeal carcinoma; OD: Optical density; PCNA: Proliferating cell nuclear antigen; PVDF: Polyvinylidene difluoride; qRT-PCR: Quantitative reverse transcriptase PCR; RNAi: RNA interference; SATB1: Special AT rich sequence binding protein 1; SDS-PAGE: Sodium dodecyl sulfate polyacrylamide gel electrophoresis; siRNA: Small interfering RNA.

## Competing interests

The authors declare that they have no competing interests.

## Authors’ contributions

ZS participated in the design of the study, analyzed the data, and drafted the manuscript. YZ and YW collected the clinical samples and participated in the design of the study. JG participated in the design of the study and drafted the manuscript. XJ, RD and CW performed the experiments. RL sectioned the clinical samples. BL and CZ scored the immunohistochemistry. HJ and WJ conceived and coordinated the study. All the authors read and approved the final manuscript.

## Supplementary Material

Additional file 1 Primer pairs used for PCR. Click here for file

Additional file 2: Figure S1 Knockdown of SATB1 in NPC cells lead to changes of EMT marker genes. Click here for file
