# Supplementary material for: Over-expression of the special AT rich sequence binding protein 1 (SATB1) promotes the progression of nasopharyngeal carcinoma: association with EBV LMP-1 expression
Source: J Transl Med. 2013 Sep 18;11:217. doi: 10.1186/1479-5876-11-217 (PMC3850651; doi:10.1186/1479-5876-11-217)
Supplement: Additional file 1 — Primer pairs used for PCR. [file 1479-5876-11-217-S1.doc]

**Additional file 1:**

Primer pairs used for quantitative PCR:

SATB1 forward, 5′-CTGGG CTCGTATCAACACCTAT-3′,

SATB1 reverse, 5′-TAAGGACTGCTGGGCTAAAAGT-3′;

β-actin forward, 5′-TGACGTGGACATCCGCAAAG-3′,

β-actin reverse, 5′-CTGGAAGG TGGACAGCGAGG-3′.

Primer pairs used for semiquantitative PCR:

LMP-1 forward, 5′-TCCTCCTCTTGGCGCTACTG-3′,

LMP-1 reverse, 5′-TCATCACTGTGTCGTTGTCC-3′;

SATB1 forward, 5′-ACCCTGGGCTCGTATCAA-3′,

SATB1 reverse, 5′-CCATTCCTTTCAGTGGCAAT-3′;

β-actin forward, 5′-CGTGGACATCCGCAAAGAC-3′,

β-actin reverse, 5′-AAGAAAGGGTGTAACGCAACT-3′.
