# Supplementary material for: Over-expression of the special AT rich sequence binding protein 1 (SATB1) promotes the progression of nasopharyngeal carcinoma: association with EBV LMP-1 expression
Source: J Transl Med. 2013 Sep 18;11:217. doi: 10.1186/1479-5876-11-217 (PMC3850651; doi:10.1186/1479-5876-11-217)
Supplement: Additional file 2: Figure S1 — Knockdown of SATB1 in NPC cells lead to changes of EMT marker genes. [file 1479-5876-11-217-S2.doc]

**Additional file 2:**


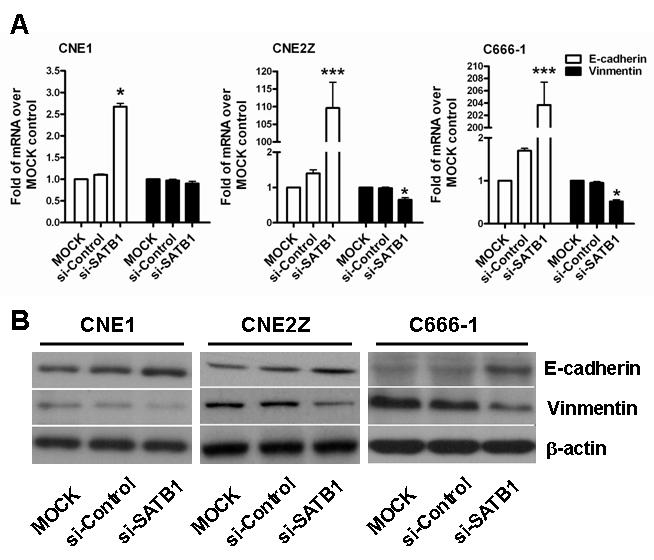


**Figure S1. Knockdown of SATB1 in NPC cells lead to changes of EMT marker genes.** Well-differentiated NPC cell line CNE1, poorly-differentiated CNE2Z and undifferentiated C666-1 cells were transfected with a cocktails of SATB1 siRNAs (si-SATB1) or negative control siRNA (si-Control) or left without transfection (MOCK) for 48 hours, **(A)**: qRT-PCR and (**B**): western blotting were used to examine the expression of marker genes of EMT (E-cadherin and Vinmentin). Data were plotted as fold change compared with the MOCK controls. * *P*< 0.05, *** *P*<0.001 vs. MOCK or si-Control.
